# Microvessel Density (Chalkley Method) in a Series of 79 Gastrointestinal Stromal Tumors

**DOI:** 10.4021/gr373w

**Published:** 2011-11-20

**Authors:** Luiz Eduardo Waengertner, Luise Meurer, Marcelle Reesink Cerski

**Affiliations:** a Programa de Pos Graduacao, Ciencias em Gastroenterologia, RS, Brazil; b Servico de Patologia do Hospital de Clinica de Porto Alegre, RS, Brazil; c Departamento de Patologia da Universidade Federal do Rio Grande do Sul, RS, Brazil

**Keywords:** GISTs, Angiogenesis, Microvessel density, Chalkley method

## Abstract

**Background:**

Evaluation of the MVD (modified Chalkley method) in a series of 79 cases of GISTs diagnosed by the Pathology Service at the HCPA (Hospital de Clinicas de Porto Alegre) from January 1993 to December 2009.

**Methods:**

Seventy nine cases of GISTs were submitted to immunohistochemical analysis for CD31, an endothelial marker, to analyze MVD. Hot spots were identified for each case, and the mean numbers of stained blood vessels collected through Chalkley count, with the use of a 25 point grid, placed onto a scanned image. Images were analysed through an image analysis system. We used a cutoff of six vessels.

**Results:**

Our series was composed of 42 males and 37 females and presented an average age of 58.9 years. GISTs were predominately located in the stomach (45.6%) followed by the small intestine (38.0%). Sixty seven GISTs (84.8%) showed an average of less than six vessels stained by CD31 (MVD) and 12 (15.2%) GISTs an average of more than six vessels. A statistically significant difference was observed between survival rate of patients having GISTs with MVD of ≤ 6 vessels (mean = 2.4, CI 95%: 1.67 - 3.17) and patients having GISTs with MVD of ≥ 6 vessels (mean = 2.4, CI 95%: 1.67 - 3.17), P = 0.001. No association for MVD was observed related to sex, age, histological type, risk category, location and metastasis.

**Conclusions:**

Seventy nine cases of GISTs diagnosed at a single center in South Brazil were studied for MVD (Chalkley method). There was a statistically significant difference between MVD and the survival rate for these patients. The use of Chalkley method in GISTs may be helpful to evaluate clinical outcome.

## Introduction

Gastrointestinal stromal tumors (GISTs) are the most common stromal tumors of the gastrointestinal tract, with an annual incidence of nearly 2/100,000/year [1-4]. They affect males and females similarly and most patients are between 50 and 60 years of age [1-5]. GISTs represent 2% of all gastric tumors, 14% of small intestine tumors and 0.5% of colonic tumors [5]. They are thought to arise from interstitial cell of Cajal, a pacemaker cell found in the myoenteric plexus [6-7]. Macroscopically, GISTs are non-encapsulated, well-defined, intra-abdominal nodular lesions, which can cause a bulging in the lumen of the gastrointestinal tract [3-8]. On histology, about 70% of GISTs are composed of spindle-cells, while epithelioid cells comprise a further 20% and remaining 10% of tumors are of mixed cell types [2, 8-11].

The tyrosine kinase receptor, CD117, is present in 90% to 95% of GISTs, usually with diffuse cytoplasmic expression [12-16]. The protein is considered the main diagnostic marker for GISTs, along with CD34, a hematopoietic stem cell marker, present in up to 70% of GISTs [5, 17-20]. Some authors, notable Fletcher et al have reported that lesion size and mitotic count can be important predictors of GISTs potential malignancy [10, 11, 13, 14, 17, 21, 22]. These characteristics are used to classify tumors into different risk categories for aggressive behavior [3, 10, 11, 17, 22].

Angiogenesis, formation of new blood vessels, plays a central role in cancer survival, local tumor growth and development of distant metastasis [23-25]. Tumor blood supply is directly related to an imbalance between pro-angiogenic and anti-angiogenic factors [26-27]. The mainstay of the assessment of tumor vascularity has been counting the number of immunohistochemical identified microvessels in vascular hot spots [27]. Microvessel density (MVD) has been studied as a prognostic marker in different kinds of human cancer [24, 28-30]. Techniques including Chalkley counting, vascular grade and the use of image analysis systems are described to evaluate angiogenesis [27], Dornelles et al measured angiogenesis using a method combining MVD, Chalkley grid and image analysis systems [31].

## Materials and Methods

**Figure 1:**
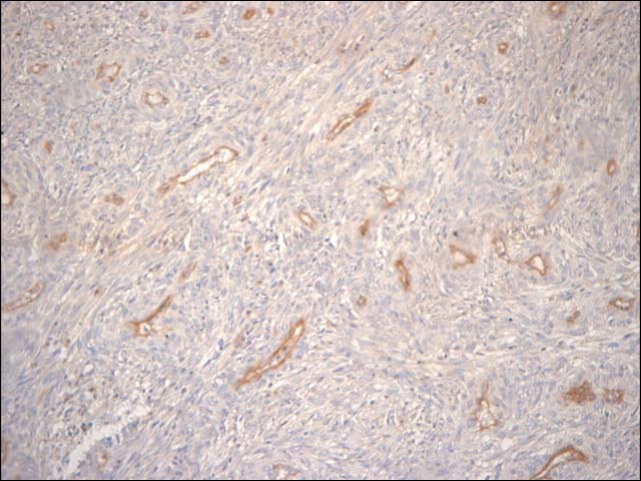
CD31 antibody in GIST (200 x).

Seventy-nine cases of GISTs diagnosed at the Department of Pathology of Hospital de Clinicas de Porto Alegre from January 1993 to December 2009 were submitted to immunohistochemistral analysis for CD31, (1:10 by DAKO), an endothelial marker, for MVD analysis. Procedures were made according to manufacturer’s instructions. For antigenic recovery we used citrate buffer with pH 6.0 and microwave oven. Initially, 3 to 5 microscopic fields (200 x) showing the highest microvascular density (hot spots) were identified, with the use of CD31 antibody (Fig. 1). The mean number of stained blood vessels was collected through Chalkley count, where a 25-point grid was placed onto a scanned image and all points coincided with the marked vessels were counted. Three to five images were used and the mean value was obtained with the number of counted vessels in each image [31].

Clinical information and follow-up were obtained from medical records of Hospital de Clinicas de Porto Alegre: age, mounth/year of diagnosis, tumor location. In the retrospective follow-up analysis we looked for local recurrence, metastases, site of metastases, use of adjuvant therapy with tyrosine kinase inhibitors, date of the last medical appointment and death as a consequence of disease activity or other causes.

## Results

### Demographic data

The sample composed of 79 cases, 42 males and 37 females presented an average age of 58.9 years ± 13. Forty patients were over 60. All our cases were considered sporadic GISTs. Eleven cases (13.9%) were described as an incidental finding during surgery. GISTs were predominately located in the stomach (45.6%) followed by the small intestine (38.0%) and (26.4%) were Iocated in omentum and mesenterium. We have not identified any case of esophageal GIST.

### Histological findings

Spindle cell morphology was present in 72.2% of these tumors. Tumor size varied, ranging from 0.5 cm to 25.0 cm, with median of 4.8 cm. According to the NIH classification, 15.4% GIST were classified as very low risk category, 13.8% low risk category, 23.1% intermediate risk category and 47.7% belonged to the high risk category.

### Immunohistochemical findings and statistical analysis

**Figure 2:**
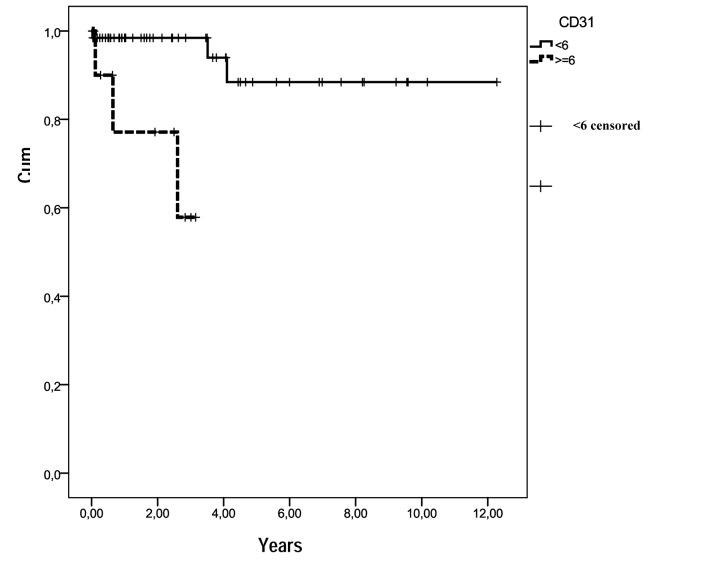
Survival functions in relation to MVD (CD31). Out of the 12 patients with MVD ≥ 6 vessels, 3 died (25.0%) and out of 67 with MVD ≤ 6 vessels, 3 died (4.5%).

CD117 was strongly expressed in 78 cases. Only one case was negative for CD117, but strongly positive for CD34, and negative for S-100 protein, desmin and actin (previous data not published). Microvessel density evaluation, through Chalkey method using scanned images, showed an average of less than six vessels (stained by the anti-CD31 antibody reaction) in 67 cases (84.8%), and an average of more than six vessels in 12 cases (15.2%). Out of 12 patients with a mean of MVD ≥ 6 vessels, 3 died (25.0%) and out of 67 patients having a mean of MVD ≤ 6 vessels, 3 died (4.5%). A statistically significant difference was seen when these findings were related to survival rates: MVD ≤ 6 vessels (mean = 2.4, CI 95%: 1.67 - 3.17) and MVD ≥ 6 vessels (mean = 2.4, CI 95%: 1.67 - 3.17), P = 0.001 (Fig. 2). No association for MVD was observed related to sex, age, histological type, risk category, location and metastasis.

### Follow-up

Our patients were followed for a mean time of 2.5 ± 2.8 years (median 1.5 years). Nine patients developed metastasis, five involving liver and four peritoneal cavity. Three patients received adjuvant therapy with tyrosine kinase inhibitors for a period not longer then 3 months.

## Discussion

Seventy-nine cases of GISTs from a single center in South Brazil were analysed for MVD to evaluate angiogenesis. Our series was composed of 79 cases, 42 males and 37 females with an average age of 58.9 years ± 13. GISTs were predominately located in the stomach followed by the small intestine, omentum and mesenterium. 15.4% GISTs were classified as very low risk category, 13.8% low risk category, 23.1% intermediate risk category and 47.7% high risk category (NIH classification).

Our patients were followed for a mean time of 2.5 ± 2.8 years (median 1.5 year). Many cases were diagnosed and added to this study in the last three years, thus impairing follow-up time. Only nine cases (11.4%) have progressed to metastasis, involving liver and peritoneal cavity. Other GISTs series with a longer follow-up time, observed metastases in 27.0% to 54.0% of their cases. The low incidence of metastasis in our series is probably related to the short follow-up time [16]. Only three patients where treated with anti-molecular therapy, 400 mg/daily, for a period not longer than 3 months, considered insufficient [32].

A high MVD may indicate poor prognosis in different kinds of human neoplasias such as prostate carcinoma, adult astrocytoma, gastric and breast cancer. These same correlation was not observed for lung and bladder cancers and cerebellar medulloblastoma [24, 29, 30, 33, 34].

MVD appeared to be an important independent factor of poor prognosis by multivariate analysis for adult astrocytomas (P = 0.001) [32]. The study of MVD in renal cell carcinoma suggested that for these tumors, MVD was inversely associated with micro vascular invasion, metastasis and patient survival [35]. Another series of 67 gastric cancer samples showed a significantly association between high MVD, and poor survival [24].

Imamura et al investigating angiogenesis in 95 GISTs, evaluated MVD through CD31 immunochemicalstaining. In his series multivariate analysis identified MVD and tumor grade as being two independent factors of worse prognosis (P = 0.0007, 0.0152 respectively), suggesting that the study of MVD may be a useful predictor of aggressive biologic behavior for GISTs [23].

In our series microvessel density evaluation, through Chalkley method using scanned images, showed an average of less than six vessels (stained by the anti-CD31 antibody reaction) in 67 cases (84.8%) and an average of more than six stained vessels in 15.2%. Out of 12 patients with a mean of MVD ≥ 6 vessels, 3 died (25.0%) and out of 67 patients having a mean of MVD ≤ 6 vessels, 3 died (4.5%). A statistically significant difference was seen when these findings were related to survival rates: MVD ≤ 6 vessels (mean = 2.4, CI 95%: 1.67 - 3.17) and MVD ≥ 6 vessels (mean = 2.4, CI 95%: 1.67 - 3.17), P = 0.001. Histological quantification of tumor vascularity may be a significant prognosticator in GISTs. No association for MVD was observed related to sex, age, histological type, risk category, location and metastasis in the present work.

Angiogenic activity may be measured by MVD, but other factors such as the vascular endothelial growth factor (VEGF), VEGF receptors (VEGFR-1, VEGFR-2), cell adhesion molecules, proteases and other cytokines markers may also be involved in the process. Future analysis for more information on the biology of tumor angiogenesis may be necessary [26, 27, 30].

### Conclusion

Seventy-nine cases of GISTs diagnosed at a single center in South Brazil were analyzed for MVD (Chalkley method). There was a statistically significant difference between MVD and survival rates for these patients. The use of Chalkley method in GISTs may be helpful to evaluate clinical outcome.
